# Ethanol Enhances Hyperthermia-Induced Cell Death in Human Leukemia Cells

**DOI:** 10.3390/ijms22094948

**Published:** 2021-05-06

**Authors:** Mercedes Quintana, Ester Saavedra, Henoc del Rosario, Ignacio González, Inmaculada Hernández, Francisco Estévez, José Quintana

**Affiliations:** Departamento de Bioquímica y Biología Molecular, Fisiología, Genética e Inmunología, Instituto Universitario de Investigaciones Biomédicas y Sanitarias (IUIBS), Universidad de Las Palmas de Gran Canaria, 35016 Las Palmas de Gran Canaria, Spain; mercedes.quintana101@alu.ulpgc.es (M.Q.); ester.saavedra102@alu.ulpgc.es (E.S.); henoc.del101@alu.ulpgc.es (H.d.R.); ignacio.gonzalez@ulpgc.es (I.G.); servanda.hernandez@ulpgc.es (I.H.); francisco.estevez@ulpgc.es (F.E.)

**Keywords:** ethanol, hyperthermia, leukemia cells, apoptosis, HSP70

## Abstract

Ethanol has been shown to exhibit therapeutic properties as an ablative agent alone and in combination with thermal ablation. Ethanol may also increase sensitivity of cancer cells to certain physical and chemical antitumoral agents. The aim of our study was to assess the potential influence of nontoxic concentrations of ethanol on hyperthermia therapy, an antitumoral modality that is continuously growing and that can be combined with classical chemotherapy and radiotherapy to improve their efficiency. Human leukemia cells were included as a model in the study. The results indicated that ethanol augments the cytotoxicity of hyperthermia against U937 and HL60 cells. The therapeutic benefit of the hyperthermia/ethanol combination was associated with an increase in the percentage of apoptotic cells and activation of caspases-3, -8 and -9. Apoptosis triggered either by hyperthermia or hyperthermia/ethanol was almost completely abolished by a caspase-8 specific inhibitor, indicating that this caspase plays a main role in both conditions. The role of caspase-9 in hyperthermia treated cells acquired significance whether ethanol was present during hyperthermia since the alcohol enhanced Bid cleavage, translocation of Bax from cytosol to mitochondria, release of mitochondrial apoptogenic factors, and decreased of the levels of the anti-apoptotic factor myeloid cell leukemia-1 (Mcl-1). The enhancement effect of ethanol on hyperthermia-activated cell death was associated with a reduction in the expression of HSP70, a protein known to interfere in the activation of apoptosis at different stages. Collectively, our findings suggest that ethanol could be useful as an adjuvant in hyperthermia therapy for cancer.

## 1. Introduction

Cancer has one of the highest worldwide mortality indices of any disease. Treatment includes chemotherapy, radiotherapy, and immunotherapy but despite the efforts and efficacy of these strategies mortality rates are still very high [1,2]. Most anticancer drugs and radiotherapy cause the death of sensitive cells by inducing apoptosis [3]. This kind of cell death can occur with or without the activation of caspases, a family of aspartate specific cysteine proteases which are generally synthesized as zymogens and activated by proteolytic cleavage [4]. There are two major caspase activation pathways [5]. The extrinsic pathway involves activation of cell surface death receptors Fas, death receptors 4 and 5 (DR4/DR5) or tumor necrosis factor receptor (TNFR) and is dependent on the initiator caspase-8 which cleaves and activates the downstream effector caspases (caspase-3, -6 and -7), inducing a cascade of caspases. The intrinsic pathway involves cytochrome c release and the subsequent activations of caspase-9 and caspase-3 [6].

Resistance is one of the major limitations in chemotherapy. Therefore, the search for new strategies and/or compounds with antitumoral properties that may increase the efficacy of the current chemotherapeutic agents is of great interest [7,8,9]. Hyperthermia is a promising alternative for cancer treatment that is based on increasing the temperature (40–43 °C) specifically in tumor tissue and the therapeutic effect depends on the strength and treatment duration [10]. A large number of experimental studies have also reported that hyperthermia increases the effectiveness of conventional antineoplastic therapies [11,12,13]. It is known that the architecture of the vasculature in solid tumor tissues is complex, with a hypoxic and acidic microenvironment [14]. In this scenario, cancer cells are highly sensitive to hyperthermia treatment and most of the healthy cells around tumor tissues are not damaged [15]. Hyperthermia induces the unfolding and aggregation of proteins, damage of the cell membrane, and disruption of cell cytoskeleton [16]. Although this treatment has shown low toxicity, mild side effects, effectiveness in killing cancer cells and apoptosis inducing properties, the acquisition of thermotolerance is a main clinical problem [17]. This has been generally associated with the synthesis and accumulation of pro-survival heat shock proteins, especially HSP70, among other factors [18]. Consequently, the possibility to reduce thermotolerance by using low toxicity compounds is of great clinical importance. In this regard, several studies in vivo and in vitro have explored non-toxic enhancers for hyperthermia-induced cell death in a variety of human cancer cells including lung cancer [19,20], colon cancer [21,22], melanoma [23], and leukemia cells [24,25,26,27].

Ethanol is a natural compound known to be primarily metabolized by liver enzymes including alcohol dehydrogenase, cytochrome P450 2E1, and microsomal ethanol-oxidizing system. All pathways that metabolize ethanol lead to the formation of acetaldehyde which may favor stress oxidative via reactive oxygen species generation [28] and is responsible, at least in part, of the widely known hepatotoxic effects. It has been reported that ethanol enhances susceptibility of human leukemia cells to apoptotic cell death [29], potentiates TRAIL-induced apoptosis in colon, leukemic T-lymphocytes, and prostate cancer cells [30,31,32], enhances genistein-induced apoptosis on human cervix cancer cells [33], and increases the toxicity of ionizing radiation in hepatocarcinoma cells [34]. However, little is known about its properties as a chemosensitizer agent in combination with hyperthermia. The aim of this study was to investigate the influence of ethanol on hyperthermia-induced cell death in human leukemia cells, to determine its impact on the intrinsic- and extrinsic- apoptotic pathways and on Bcl-2 family members’ expression. We found evidence that non-toxic concentrations of this alcohol enhance hyperthermia-induced apoptotic cell death which was related with a decrease in HSP70 levels.

## 2. Results

### 2.1. Ethanol Increases Hyperthermia-Induced Cell Death in Human Myeloid Leukemia Cells

**Figure 1:**
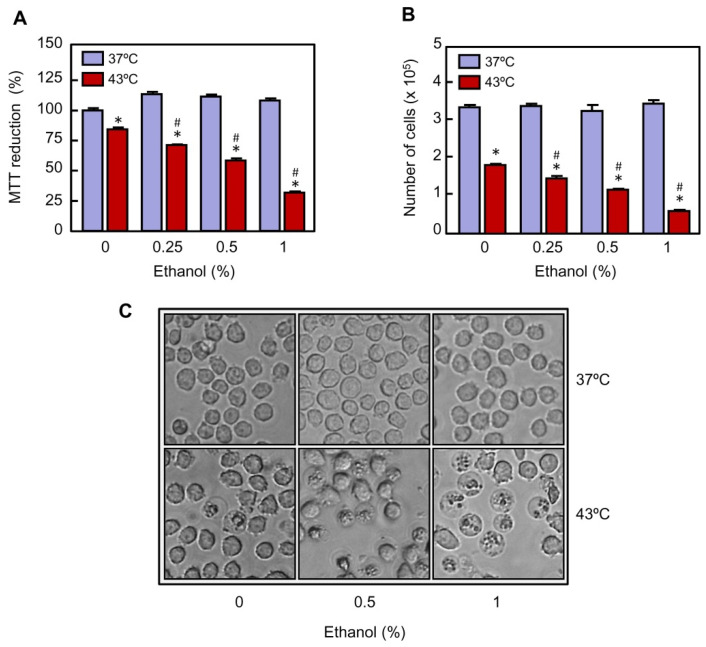
Effect of ethanol on cell viability in U937 cells exposed to hyperthermia. The cells were pre-incubated in absence or presence of the indicated concentrations of ethanol for 30 min, exposed to hyperthermia (30 min at 43 °C) and allowed to recover for 24 h. (**A**) Mitochondrial activity was evaluated by the MTT reduction assay. (**B**) The number of viable cells was determined by the trypan blue exclusion method using an automatic cell counter. (**C**) The cells were visualized under phase contrast microscopy; a representative field of each experimental group is shown. Magnification 20×. * *p* < 0.05 vs. respective control at 37 °C; # *p* < 0.05 vs. hyperthermia (43 °C) treatment alone.

U937 cells are a widely used model to study the heat-induced response against chemical and physical stimuli since they exhibit a quick and robust response. To study the effect of ethanol on U937 cells exposed to hyperthermia a range of nontoxic concentrations was used. To this end, the cells were cultured in absence or presence of ethanol (0.25%, 0.5%, and 1%) for 1 h and then the cells were subjected to mild hyperthermia (30 min at 43 °C). The cells were harvested and analyzed following a 24-h recovery period at 37 °C. As shown (Figure 1A), a clear decrease in the MTT reduction was observed in the cells exposed to hyperthermia alone and the decrease in mitochondrial activity was more evident in the cells exposed to hyperthermia in combination with increasing concentrations of ethanol. Therefore, the lower mitochondrial activity, approximately 25% of control values, was observed in the group treated with hyperthermia in combination with 1% ethanol. Because a decrease in the percentage of MTT reduction may indicate inhibition of cell proliferation, increase in cell death, or both, the effect of ethanol on the cell number was also evaluated by the trypan blue exclusion method. As shown (Figure 1B), the number of viable cells decreased in response to hyperthermia (~57% of viable cells as compared to control), and the increasing concentrations of ethanol greatly augmented the efficacy of hyperthermia (~10% of viable cells as compared to control in the cells treated with 1% ethanol). The results also demonstrated that ethanol by itself did not have any effect on the number of cells. Consistent with the above results, when U937 cells were cultured with ethanol at 37 °C, they appeared to be healthy as visualized under phase contrast microscopy after a 24-h period of recovery (Figure 1C). In contrast, combination of hyperthermia and ethanol significantly augmented the number of unhealthy cells in comparison with hyperthermia alone. Taken together, these results suggest that ethanol potentiates the cytotoxicity of hyperthermia in U937 cells.

**Figure 2:**
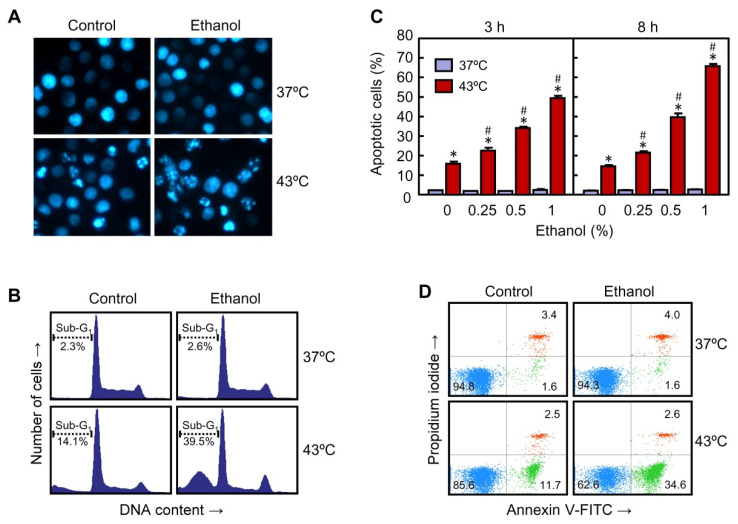
Effect of ethanol on apoptotic cell death in U937 cells subjected to hyperthermia. (**A**) The cells were pre-incubated in absence or presence of 0.5% ethanol for 30 min, exposed to hyperthermia (30 min at 43 °C) and allowed to recover for 24 h. The cells were collected, stained with bisbenzimide trihydrochloride and nuclei visualized by fluorescent microscopy; photomicrographs of representative fields of cells to evaluate nuclear chromatin condensation (i.e., apoptosis) are shown. Magnification 40×. (**B**) The cells were pre-incubated in absence or presence of 0.5% ethanol for 30 min, exposed to hyperthermia (30 min at 43 °C) and allowed to recover for 8 h. The cells were harvested and cell distribution, according to their DNA content, was determined by flow cytometry using the propidium iodide staining procedure; the position of cells with sub-G1 DNA content (i.e., apoptotic cells) is indicated by a dotted line. Representative histograms are shown. (**C**) The cells were pre-incubated in absence or presence of the indicated concentrations of ethanol and allowed to recover for the indicated period of time. The cells were collected, and the percentage of apoptotic cells was determined by flow cytometry as above. (**D**) The cells were pre-incubated in absence or presence of 0.5% ethanol for 30 min, exposed to hyperthermia (30 min at 43 °C) and allowed to recover for 3 h. The cells were collected, stained with propidium iodide and annexin V-FITC and analyzed by flow cytometry. * *p* < 0.05 vs. respective control at 37 °C; # *p* < 0.05 vs. hyperthermia (43 °C) treatment alone.

Hyperthermia is a recognized apoptosis inducer in a wide variety of human tumor cells. To know the influence of ethanol on hyperthermia-induced apoptosis, U937 cells were pre-incubated with 0.5% ethanol and then exposed to heat treatment. As shown in Figure 2A, hyperthermia alone induced morphological changes in the nuclei like condensed and fragmented chromatin, characteristic of apoptotic cells, and the number of apoptotic nuclei augmented in cells treated with hyperthermia plus ethanol. Similar results were observed when the cells were analyzed by flow cytometry after staining with propidium iodide. The histograms (Figure 2B) reveal an increase in the proportion of cells with sub-G_1_ DNA content (i.e., apoptotic cells) in the hyperthermia plus ethanol group in comparison with hyperthermia alone (39.5% vs. 14.1%) following an 8-h recovery period. The effect of increasing concentrations of ethanol on hyperthermia-induced apoptosis was evaluated and quantified at different time periods of recovery (Figure 2C). A clear increase in the percentage of apoptotic cells was observed early (3 h) in the hyperthermia plus ethanol combination groups in comparison with hyperthermia alone. The maximal level of apoptotic cells (3-fold increase) was achieved with the highest concentration of ethanol used (1%). Similar levels of potentiation were also observed when cells were allowed to recover for longer periods of time (8–24 h). In accordance with the results showed in Figure 1, treatment with ethanol (0.25–1%) at 37 °C did not have any impact on the number of apoptotic cells (Figure 2C). Double staining of the cells with propidium iodide and annexin V-FITC and analysis by flow cytometry revealed an increase in the percentage of annexin V-positive and propidium iodide-negative cells (i.e., apoptotic cells) in response to hyperthermia (11.7%) or hyperthermia plus ethanol (34.6%) as compared with the respective controls (1.6%) (Figure 2D). The increase in the percentage of cells in the right/lower quadrant in the group hyperthermia plus ethanol in comparison to hyperthermia treated alone (3-fold, 34.6% vs. 11.7%) was similar to the increase in the percentage of cells in the sub-G1 fraction of the cell cycle (Figure 2B,C). It was not detected an increase in the number of primary necrotic cells or late apoptotic/secondary necrotic cells in response to hyperthermia alone (2.5% vs. 3.4%) or hyperthermia plus ethanol (2.6% vs. 4.0%), as observed in the right/upper quadrants (Figure 2D), suggesting that necrotic cell death modality is not activated.

**Figure 3:**
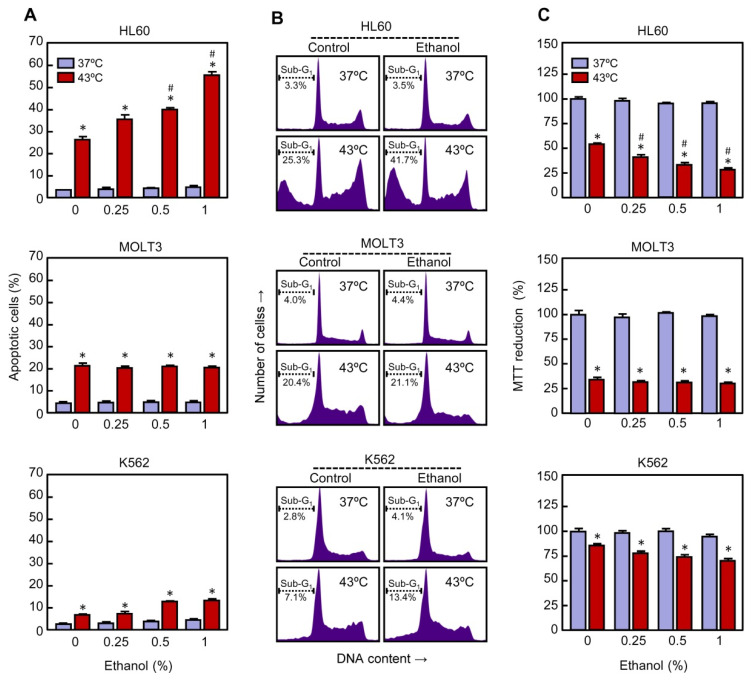
Effect of ethanol on apoptotic cell death in human leukemia cell lines subjected to hyperthermia. The cells were pre-incubated in absence or presence of ethanol for 30 min, exposed to hyperthermia (30 min at 43 °C), and allowed to recover for the indicated period of time. (**A**) The cells were collected after a 3 h period of recovery and cell distribution according to their DNA content was determined by flow cytometry, using the propidium iodide staining procedure; percentage of apoptotic cells (i.e., fraction of cells with sub-G_1_ DNA content) is shown. (**B**) The cells were allowed to recover for 3 h and cell distribution according to their DNA content was determined by flow cytometry, as above; the position of apoptotic cells is indicated by a dotted line. Representative histograms are shown. (**C**) The cells were collected following a 24 h period of recovery and cytotoxicity evaluated by the MTT reduction method. * *p* < 0.05 vs. the respective control at 37 °C; # *p* < 0.05 vs. hyperthermia (43 °C) treatment alone.

To determine whether the potentiation of cell death by ethanol in U937 cells subjected to hyperthermia is also observed in other human leukemia cells, the cell lines HL60 (acute myeloid cells), K562 (chronic myeloid cells), and MOLT3 (acute T lymphoblastic cells) were included in this study. These cells were pre-incubated in the absence or presence of ethanol, treated with mild hyperthermia and, following a recovery period specified for each cell line, analyzed by flow cytometry using the propidium iodide staining procedure. As shown (Figure 3A), the percentage of apoptotic cells increased in HL60 in response to hyperthermia as compared to control (25% vs. 4%) and ethanol (0.25–1%) enhanced the efficacy of this treatment in a similar way as observed in U937 cells. With the higher concentration of ethanol tested (1%) a 2-fold increase in the percentage of apoptotic cells was achieved in comparison with hyperthermia-treated cells alone (52% vs. 25%).

The apoptotic machinery is also activated by heat in MOLT3 cells; an increase in the number of apoptotic cells was observed after treatment with hyperthermia alone as compared to unheated cells (22% vs. 5%). In contrast, ethanol (0.25–1%) had no influence on hyperthermia-induced apoptosis in these cells. We also include in this study K562 cells because they are highly resistant to hyperthermia-induced apoptosis [35]. These cells were treated as above and they were allowed to recover for 24 h. As expected, the fraction of apoptotic cells increased slightly in response to hyperthermia as compared to untreated cells (7% vs. 3%). When ethanol was included (0.25–1%) the efficacy of hyperthermia in inducing apoptosis was not improved. Representative histograms on the effect of ethanol on hyperthermia-induced apoptosis in HL60, MOLT3, and K562 are shown (Figure 3B). Consistent with the above results, the study on cell viability using the MTT reduction procedure indicates that ethanol, in a concentration dependent manner, enhanced the cytotoxicity of mild hyperthermia in HL60 but not in MOLT3 or K562 cells (Figure 3C). Together, the results on leukemia cells indicate that in the conditions and time frame evaluated, ethanol by itself did not show significant effect on apoptosis and cell viability; however, it differentially modulated the apoptotic response to hyperthermia.

### 2.2. Ethanol Increases Processing and Activity of Caspases in Hyperthermia Treated Cells

**Figure 4:**
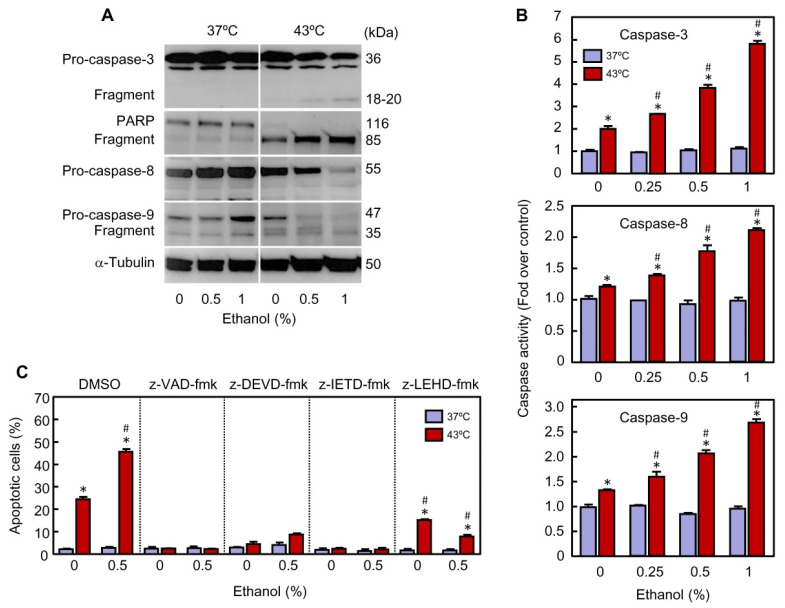
Effect of ethanol on processing and activity of caspases in U937 cells subjected to hyperthermia. (A–C) The cells were pre-incubated in absence or presence of the indicated concentrations of ethanol for 30 min, exposed to hyperthermia (30 min at 43 °C) and allowed to recover for 3 h. (**A**) Cleavage of indicated pro-caspases was analyzed by immunoblotting; as a loading control α-tubulin was also determined. (**B**) The activity of specified caspases was determined from lysates using colorimetric substrates and results are expressed as fold increase in caspase activity compared with the respective control. (**C**) Effect of cell-permeable caspase inhibitors on modulation of apoptosis by ethanol in cells subjected to hyperthermia. The cells were pre-treated for 1 h in absence (vehicle, 0.1% DMSO) or presence of 100 μM of z-VAD-fmk (broad-spectrum caspase inhibitor), 50 μM z-DEVD-fmk (caspase-3 inhibitor), 50 μM z-IETD-fmk (caspase-8 inhibitor), or 50 μM z-LEHD-fmk (caspase-9 inhibitor), exposed to hyperthermia with or without ethanol, collected after the time period of recovery, and analyzed by flow cytometry using the propidium iodide staining method.* *p* < 0.05 vs. the respective control at 37 °C; # *p* < 0.05 vs. hyperthermia treatment alone (containing DMSO) or hyperthermia plus ethanol (containing DMSO).

One of the most significant molecular markers of apoptosis induction both in normal and tumor cells is the activation of caspases. To determine the role of these proteolytic enzymes in the effect of ethanol on apoptosis triggered by hyperthermia, lysates from U937 cells were assayed by immunoblotting. As shown (Figure 4A), the combination of hyperthermia plus ethanol was more effective to promote the processing of pro-caspase-3, pro-caspase-8 and pro-caspase-9 (indicative of activation) than hyperthermia alone. Activation of caspase-3 was also confirmed by cleavage of its known substrate poly(ADP)ribose-polymerase (PARP), to give a 85-kDa fragment. The enhancing effect of ethanol on hyperthermia-induced apoptosis was concentration-dependent and maximal levels of proteolytic processing of these zymogens were achieved in presence of the highest concentration of ethanol used. Incubation of the cells in presence of ethanol at 37 °C did not have any effect on the processing of pro-caspases, in accordance with the results shown in Figure 2.

To confirm that the processing of pro-caspases is associated with an increase in activity, cell lysates were assayed for the cleavage of the tetrapeptide substrates Ac-DEVD-pNA (caspase-3), Ac-IETD-pNA (caspase-8), and Ac-LEHD-pNA (caspase-9). Exposure to hyperthermia stimulated caspase-3 (2.0-fold), caspase-8 (1.3-fold) and caspase-9 (1.4-fold) activities, and ethanol improved the efficacy of hyperthermia to induce these enzymatic activities (Figure 4B). The effect of ethanol was concentration-dependent and the maximal levels of caspase activity (6-fold, 2.2-fold and 2.8-fold, for caspases-3, -8, and -9, respectively) were obtained with 1% ethanol. As expected, the increase in the activities of initiator caspases (i.e., caspase-8 and caspase-9) was significantly lower than the activity of caspase-3, the main executioner caspase. Thus, consistent with the above immunoblotting studies, incubation with ethanol (0.25–1%) alone did not exert any effect on caspase activity. Overall, these results suggest that the extrinsic (caspase-8 dependent) and the intrinsic (caspase-9 dependent) pathways of apoptosis are activated in U937 cells subjected to hyperthermia and that ethanol plays a sensitizing role by enhancing the activity of caspase-3, the main executioner caspase involved in dismantling the cells.

The role of caspases in the induction of apoptosis by hyperthermia and in the potentiation of this kind of cell death by ethanol was determined using the broad-spectrum caspase inhibitor z-VAD-fmk (100 μM). As seen (Figure 4C), pre-incubation of the cells with this inhibitor completely blocked apoptotic cell death triggered by hyperthermia, in the absence as well as in the presence of ethanol. These results suggest that the effect of ethanol on hyperthermia-induced cytotoxicity is attributable to a rise in caspase activity. To further identify which caspases were important in the potentiation of cell death by ethanol, U937 cells were pre-incubated with the cell-permeable specific caspase inhibitors z-DEVD-fmk, (caspase-3 inhibitor), z-IETD-fmk (caspase-8 inhibitor), or z-LEHD-fmk (caspase-9 inhibitor) and the number of apoptotic cells were determined by flow cytometry at 3 h of recovery. As shown (Figure 4C), caspase-3 and caspase-8 inhibitors completely abrogated apoptosis in cells subjected to hyperthermia as well as in cells exposed to hyperthermia plus ethanol. In contrast, the caspase-9 inhibitor partially blocked apoptotic cell death in cells exposed to hyperthermia (24% in absence vs. 15% in the presence of inhibitor) while the blockage was more effective in cells exposed to hyperthermia plus ethanol (45% in absence vs. 8% in presence of inhibitor). These results suggest that the activation of the extrinsic pathway of apoptosis is essential for hyperthermia induced apoptosis both in the absence and presence of ethanol. The intrinsic pathway, however, contributes partially to the heat-induced apoptosis, but it plays a crucial role in the cells exposed to hyperthermia in combination with ethanol.

### 2.3. Ethanol Increases the Release of Apoptogenic Factors from Mitochondria in Cells Subjected to Hyperthermia

**Figure 5:**
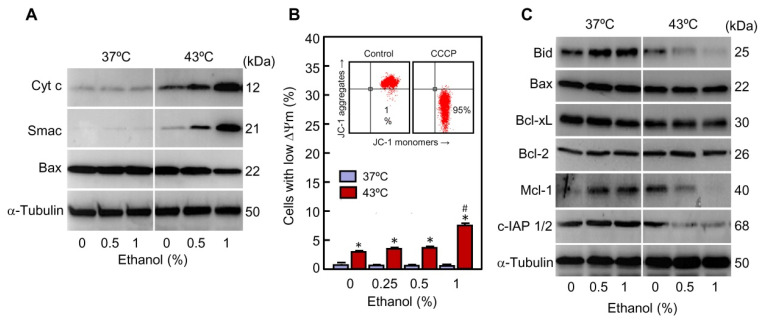
Effect of ethanol on pro-apoptotic and anti-apoptotic factors involved in the mitochondrial pathway of apoptosis in U937 cells subjected to hyperthermia. (**A**–**C**) The cells were pre-incubated in absence or presence of the indicated concentrations of ethanol for 30 min, exposed to hyperthermia (30 min at 43 °C) and allowed to recover for 3 h. (**A**) Cytosolic fractions were isolated and analyzed by immunoblotting; α-tubulin was used as a loading control. (**B**) Mitochondrial transmembrane potential (∆ψm) was evaluated by flow cytometry using the JC-1 probe (10 µM) and expressed as percentage of cells with low mitochondrial transmembrane potential (low green fluorescence); cells treated with 50 μM of CCCP for 30 min were used as a positive control (inserted dot plot). (**C**) Whole cell lysates were prepared and assayed by immunoblotting; as a loading control, α-tubulin was also determined. * *p* < 0.05 vs. the respective control at 37 °C; # *p* < 0.05 vs. hyperthermia (43 °C) treatment alone.

The release of cytochrome c from mitochondria to the cytosol is a key event to activate caspase-9. As revealed by the immunoblot assays (Figure 5A) this apoptogenic factor was slightly detected in the cytosolic fraction from cells incubated at 37 °C and basal levels were not affected by ethanol. In contrast, the levels of cytochrome c increased in response to hyperthermia alone and ethanol, in a dose-dependent manner, greatly improved the release. In agreement with the mitochondrial outer membrane permeabilization, Smac/Diablo also augmented in the cytosol of cells subjected to hyperthermia plus ethanol. This protein was hardly detected in the cytosolic fractions from control cells or from cells only treated with ethanol. It is known that Smac/Diablo contributes to activation of apoptosis, through relieves the inhibitory effect of the inhibitors of apoptosis proteins (IAPs) on caspases in the cytosolic compartment [36].

The release of intermembrane factors may be provoked by mitochondrial permeability transition, an abrupt increase in the permeability of the inner mitochondrial membrane to low molecular weight solutes. It causes dissipation of the mitochondrial transmembrane potential (Δψm) and therefore suppression of essential activities such as ATP synthesis. To determine whether the release of cytochrome c is associated with a disruption of the mitochondrial transmembrane potential, cells were stained with the fluorescent probe JC-1 and analyzed by flow cytometry. As shown in Figure 5B, a slight increase in the fraction of cells with low Δψm was observed in hyperthermia-treated cells as compared to control cells (4% vs. 1%) at 3 h of recovery. A modest increase in the percentage of cells with low Δψm was also appreciated in the group exposed to hyperthermia in combination with the highest concentration of ethanol used as compared to hyperthermia alone (7% vs. 4%). Treatment with ethanol alone (0.25–1%) had not any influence on the mitochondrial potential. In fact, although hyperthermia alone provoked maximum levels of apoptotic cells at 3 h of recovery (Figure 2B) only a reduced percentage of cells showed low Δψm at that time (Figure 5B). This suggests that the marginal Δψm dissipation observed is probably a caspase activation consequence. Cells treated with the depolarizing agent carbonyl cyanide 3-chlorophenylhydrazone (CCCP) for 30 min were included as a positive control; in these conditions >95% of cells exhibited low mitochondrial membrane potential (Figure 5B, bottom/right quadrants in the inserted dot plot).

### 2.4. Ethanol Modulates the Expression of Pro-Apoptotic and Anti-Apoptotic Members of Bcl-2-Family in Cells Exposed to Hyperthermia

Since the intrinsic pathway of apoptosis is controlled by the Bcl-2 family proteins, the expression of pro-apoptotic members Bid and Bax was also analyzed by immunoblotting (Figure 5C). Bid is a well-recognized caspase-8 substrate which is transformed into an active cleaved-form (t-Bid) that plays a key role as a link between the extrinsic and intrinsic pathways of apoptosis. The levels of full-length Bid (25 kDa) decreased slightly (which is indicative of activation) in hyperthermia-treated cells while a greater reduction was detected in the cells exposed to hyperthermia combined with ethanol. These results are in accordance with a higher increase in caspase-8 activity in the cells subjected to hyperthermia plus ethanol in comparison with the cells treated only with hyperthermia (Figure 4A,B). It is recognized that once activated, Bid quickly associates with the outer mitochondrial membrane promoting conformational changes of cytosolic Bax and Bak which then insert into the membrane to promote permeabilization [37]. Accordingly with this model, the cytosolic levels of Bax were lower in the cells exposed to hyperthermia in combination with ethanol compared to the cells treated only with hyperthermia (Figure 5A). In accordance with these results, ethanol did not change the expression of Bax (Figure 5B).

Anti-apoptotic members of the Bcl-2 family play its role by binding to BH3-only proteins to inhibit their interaction with and activation of Bax or Bak, and their expression may be influenced by apoptotic stimuli [38]. To determine whether ethanol promotes changes in the expression of these factors during hyperthermia treatment, Bcl-xL, Bcl-2 and Mcl-1 were also analyzed by immunoblotting (Figure 5C). The results revealed that levels of Bcl-xL and Bcl-2 were not modified by the treatments while the expression of Mcl-1 was clearly reduced by hyperthermia, and ethanol, in a dose dependent manner, augmented the effect of hyperthermia on the levels of this anti-apoptotic factor. The decrease of Mcl-1 is an important event in the enhancement effect of ethanol on hyperthermia-induced cell death since this protein can inhibit apoptosis activation by binding with Bax/Bak to disturb the formation of oligomers [39]. Together, these results suggest that the enhancement of cytochrome c release by ethanol in cells exposed to hyperthermia seems to be independent of mitochondrial permeability transition and involves activation of the pro-apoptotic factors Bid and Bax and downregulation of the anti-apoptotic factor Mcl-1. It is also known that apoptosis is negatively regulated by members of the IAP family of proteins which inhibit caspase activity by directly binding to the active enzymes [36,40]. It is interesting to note that ethanol in combination with hyperthermia greatly reduced the levels of c-IAP1/2, which may contribute to the increase of the percentage of apoptotic cells observed in presence of the alcohol.

### 2.5. Ethanol Must Be Present during Hyperthermia Treatment to Achieve Potentiation and to Reduce the Expression of the Cytoprotector HSP70

**Figure 6:**
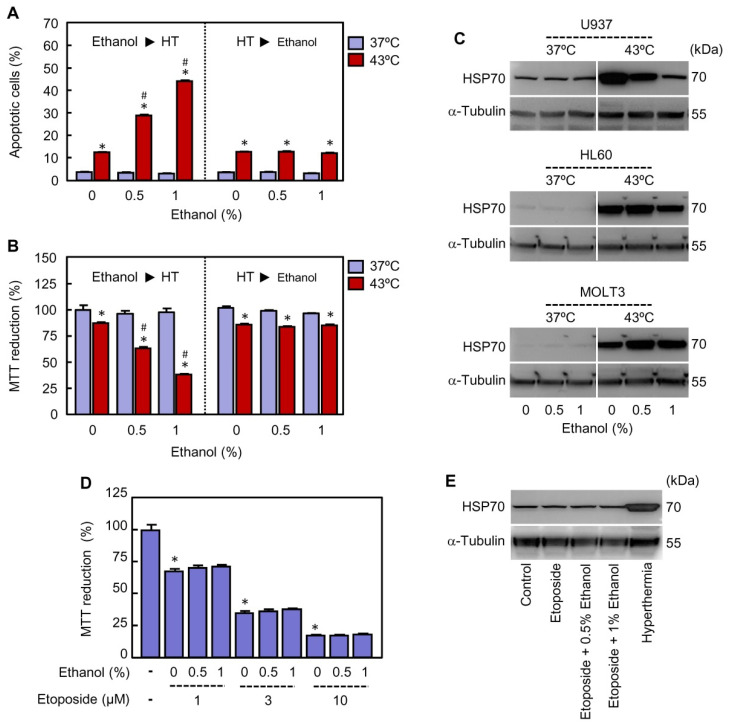
Impact of the moment of ethanol addition on modulation of cell death triggered by hyperthermia (HT) and role of HSP70. (**A**,**B**) U937 cells were treated with the indicated concentrations of ethanol before (Ethanol ► HT, left panel) or after (HT ► Ethanol, right panel) they were immersed for 30 min in a water bath at 37 °C or 43 °C, as indicated. (**A**) The cells were incubated at 37 ºC for 3 h of recovery and the percentage of apoptotic cells determined by flow cytometry using the propidium iodide staining procedure. (**B**) The cells were incubated at 37 °C for 24 h of recovery and the mitochondrial activity determined at 570 nm by the MTT reduction assay using a microplate reader. (**C**) The cells were exposed to hyperthermia (30 min at 43 °C) in presence of the indicated concentrations of ethanol and allowed to recover at 37 °C for 3 h (U937) or 8 h (HL60 and MOLT3). Whole cell lysates were prepared and levels of HSP70 analyzed by immunoblotting; as a loading control, α-tubulin was also determined. (**D**) U937 cells were incubated with etoposide (1–10 µM) in presence of the specified concentration of ethanol at 37 °C for 24 h and metabolic activity determined by the MTT reduction assay. (**E**) U937 cells were incubated with etoposide (10 µM) in absence or presence of the specified concentration of ethanol for 6 h and the expression of HSP70 determined by immunoblotting; as a loading control, α-tubulin was also included. * *p* < 0.05 vs. respective control; # *p* < 0.05 vs. hyperthermia (43 °C) treatment alone.

In all of the above described experiments, ethanol was always present during hyperthermia and recovery periods. In order to determine whether the presence of ethanol is required during the period of hyperthermia to achieve potentiation of cell death, the alcohol (0.5% or 1%) was added to the cells just before or immediately after hyperthermia treatment. As expected and in accordance with the above results, the presence of ethanol during hyperthermia period potentiates apoptotic cell death and cytotoxicity as compared to hyperthermia alone (Figure 6A,B, left panel). In contrast, whether the alcohol is added immediately after hyperthermia regime, i.e., once the cells returned to 37 °C, no effect on apoptosis stimulated by heat was detected (Figure 6A, right panel). Accordingly, differences in the reduction of MTT were not observed in the cells treated with ethanol after hyperthermia treatment compared with the cells exposed to hyperthermia alone (Figure 6B, right panel). Together, these results suggest that the presence of ethanol during hyperthermia treatment (30 min) is critical for the potentiation of heat-induced cytotoxicity.

It is widely known that hyperthermia is a potent inducer of stress response characterized by the stimulation of heat shock proteins expression, including HSP70. Since this protein plays an important role as cell death modulator via interaction to several components of the apoptotic pathways [41], the impact of ethanol on the expression of HSP70 was determined by immunoblotting in ethanol-sensitive (U937 and HL60) and ethanol-resistant (MOLT3) cells. As shown (Figure 6C), hyperthermia increased the basal levels of HSP70 in the three cell lines analyzed and ethanol blocked in different extension the expression of this protein in both U937 and HL60 cells. In contrast, HSP70 induction by hyperthermia treatment was not abrogated by ethanol in MOLT3 cells. To further confirm the importance of HSP70, U937 cells were treated with etoposide (1–10 µM), an antitumoral agent which is known to induce cytotoxicity through a mechanism independent of HSP70 upregulation [42]. The results revealed that ethanol (0.5–1%) neither enhances cytotoxicity (Figure 6D) nor modifies the levels of HSP70 (Figure 6E) in cells treated with etoposide; as a positive control, in a parallel experiment the cells were also exposed to hyperthermia alone (30 min at 43 °C) and HSP70 was analyzed by immunoblotting (Figure 6E).

## 3. Discussion

Hyperthermia is a realistic treatment option against cancer since temperatures in the range of 40–43 °C can reduce proliferation and induce cell death. It has been used for cancer treatment in combination with chemotherapy or radiotherapy [43,44]. There are several physical approaches for inducing hyperthermia, including electromagnetic radiation, ultrasound, hyperthermic perfusion, and conductive heating [10]. Nanoparticles with appropriate external energy sources are being used for local treatment [45,46,47]. Strategies to enhance the therapeutic efficacy of hyperthermia are also under investigation; in this context cytotoxic properties of hyperthermia on leukemia, melanoma, lung carcinoma, and colon cancer cells, among others, have been improved by the use of compounds that sensitize to the cells [19,20,21,22,23,24,25,26,27]. In the present study the effect of ethanol on cytotoxicity exhibited by hyperthermia on leukemia cells was investigated since this alcohol has been reported to enhance the antitumor properties of radiation and the effectiveness of chemotherapeutic agents like TRAIL [29,30,31,32,34]. A large number of studies have described that hyperthermia decreases cell viability mainly through induction of apoptosis [14,48,49,50]. The results presented herein demonstrate that ethanol enhances apoptotic cell death in U937 and HL60 leukemia cells exposed to hyperthermia. The sensitization effect of the alcohol on U937 cells was detected at 3 h of recovery and it lasted at least 24 h. Previous studies have reported that ethanol itself may reduce proliferation of cancer cells by mechanisms that involve apoptosis and that usually require long incubation times [51,52]. In our setting, ethanol alone neither induces apoptosis nor promotes cell growth arrest even at the longer period of recovery analyzed (24 h).

It has been reported that ethanol under serum starvation conditions may stimulate apoptosis via autophagy inhibition [29]. Although we have observed a marginal increase in the levels of the autophagy marker beclin-1 by hyperthermia, the amount of this protein was not affected by ethanol. It suggests that this alcohol promotes cytotoxicity of hyperthermia via an autophagy independent mechanism. In accordance with these results, the autophagy inhibitor 3-methyladenine (5 mM) had not any influence on apoptosis in hyperthermia- or in hyperthermia plus ethanol-treated cells (Supplementary Materials, Figure S1).

The immunoblotting and the enzymatic studies on cell lysates revealed a significant increase in the processing and activity of caspase-3, caspase-8, and caspase-9 in cells exposed to hyperthermia plus ethanol as compared to hyperthermia alone at 3 h of recovery period. To delineate the role of caspases in the effect of ethanol on hyperthermia, cells were pre-treated with specific and broad-spectrum caspase inhibitors. The results revealed that the potentiation of apoptotic cell death induced by ethanol was dependent on caspase activation since apoptosis was completely abolished by the pan-caspase inhibitor z-VAD-fmk. Specific inhibitors against caspase-3 (z-DEVD-fmk) and caspase-8 (z-IETD-fmk) also blocked completely apoptosis triggered by hyperthermia and suppressed the effect of combination with ethanol. These results suggest that under our experimental conditions, apoptotic cell death stimulated by hyperthermia involves activation of the extrinsic pathway.

The activation of caspase-8 is initiated following binding of death receptors (Fas, TNFR, DR4 or DR5) to their ligands (FasL, TNF or TRAIL) at the cell surface. It has been reported that hyperthermia stimulates Fas externalization and that the enhancement of apoptotic cell death by the combination with certain chemicals is associated with the increase of this death receptor on the cell surface on leukemia cells [26,53]. Since U937 and HL60 cells express most of death receptors and their ligands [54] further studies will be necessary to clarify their role in the potentiation of the hyperthermia-induced cell death by ethanol. Caspase-8 directly activates caspase-3 to initiate apoptosis and also cleaves Bid to an active form (tBid), enabling a crosstalk to mitochondrial pathway to amplify the apoptotic response. Many studies suggest that active Bid rapidly associates with the mitochondrial membrane, where it transiently binds and activates cytosolic Bax or Bak. Activated Bax/Bak inserts into the mitochondrial outer membrane where it oligomerizes forming a toroidal pore [55,56], allowing release of apoptogenic factors such as cytochrome c from the intermembrane space. Our results indicate that Bid hydrolysis was higher in cells treated with hyperthermia plus ethanol which is consistent with a higher activity of caspase-8 observed in this experimental group. Thus, cytochrome c release was detected in cells treated with hyperthermia and the levels of this apoptogenic factor were also higher in presence of ethanol.

The stimulation of cytochrome c release by ethanol was not associated with a decrease of the mitochondrial transmembrane potential (∆ψm). Only a small fraction of cells exhibited low ∆ψm, measured at a time at which maximal levels of apoptotic cells are detected (3 h). Thus, the marginal effect observed may represent a caspase-dependent event since under apoptotic conditions caspase-3 may cleave the p75 subunit of the mitochondrial respiratory chain complex I, leading to disruption of electron transport, dissipation of ∆ψm and finally reduction of ATP biosynthesis [57]. Previous studies have also shown that mitochondrial potential dissipation is not required for the complete release of cytochrome c upon mitochondrial outer membrane permeabilization [58].

It is well established that cytosolic cytochrome c engages apoptotic protease-activating factor 1 (Apaf1) and induces its oligomerization, leading to apoptosome formation and caspase-9 activation [40]. The relevance of the mitochondrial pathway in the hyperthermia-induced apoptotic cell death amplification by ethanol was analyzed by using a specific inhibitor against caspase-9 (z-LEHD-fmk). In this condition, the percentage of apoptotic cells due to hyperthermia alone decreased slightly in presence of the inhibitor while a greater inhibitory effect was appreciated when ethanol was present during hyperthermia treatment. Apoptogenic factors others than cytochrome c may be released from mitochondria to modulate the apoptotic response. In this context, cytosolic Smac/Diablo, which is known to neutralize XIAP and other IAPs [36,59] to derepress effector caspase-3, augmented greatly in cells exposed to hyperthermia plus ethanol as compared to hyperthermia treatment alone. In addition, the expression of c-IAP 1/2 decreased markedly by the combined treatment which is in accordance with previous studies indicating that Smac/Diablo may selectively reduce the protein levels of c-IAP1/2 through the ubiquitin/proteasome pathway [60]. Together, these results suggest that although caspase-8 activation is an obligatory step in hyperthermia-induced apoptosis, ethanol switches to the mitochondrial pathway to induce cell death by hyperthermia. This crosstalk between the extrinsic and intrinsic pathways to amplify the number of apoptotic cells is found in so named type II cells [59,61].

The activation of the mitochondrial pathway of apoptosis is determined by the balance of pro-apoptotic (Bid, Bax, Bak, and others) and anti-apoptotic (Bcl-2, Bcl-xL, Mcl-1, and others) members of the Bcl-2 family. Alteration of this equilibrium by different pro-apoptotic stimuli shifts the balance favouring Bax/Bak activation. The expression profile of anti-apoptotic factors revealed that hyperthermia treatment reduces the levels of Mcl-1 which were drastically downregulated in the presence of ethanol. It has been reported that Mcl-1 can inhibit apoptosis activation by binding with Bax/Bak to disturb the formation of oligomers [62]. Thus, targeting Mcl-1 has been considered as a promising approach for cancer treatment since its overexpression has been widely reported in both hematological and solid tumors [39,62,63,64]. In contrast, levels of other anti-apototic factors like Bcl-2 and Bcl-xL and the pro-apoptotic factor Bax were not altered by hyperthermia—which is in agreement with previous studies [25,65,66]—and combination of hyperthermia plus ethanol did not change the expression pattern of these proteins. The total amount of Bax was not affected by ethanol; however, there was a clear reduction in its cytosolic levels, which suggests translocation to the mitochondria to promote the permeability of this organelle.

It seems clear from the above exposed results that other factors are probably involved to explain the increases in the apoptotic stimuli by ethanol in cells subjected to hyperthermia and the greater efficacy of caspase-9 inhibitor to block cell death in the combined group. It has been well established that HSP70, in addition to its recognized role as a molecular chaperone by assisting accurate folding of nascent polypeptides and misfolded proteins, can block apoptosis induction at several stages of the apoptotic machinery [41]. For example, HSP70 stabilizes the anti-apoptotic factor Mcl-1 [50] and prevents Bax translocation [67,68], interferes in the activation of stress-activated protein kinase SAPK/JNK [69], inhibits the formation of the apoptosome [70,71], neutralizes the apoptosis-inducing factor (AIF) and blocks its nuclear import [72] and may inhibit caspase-3 activity [73,74]. It also has been reported that HSP70 inhibits TRAIL-induced DISC assembly in human acute leukemia cells, blocking therefore the activation of the extrinsic pathway [35]. Our results are in consonance with the recognized anti-apoptotic role of HSP70. Ethanol blocked HSP70 expression and increased the mitochondrial release of cytochrome c and Smac/Diablo triggered by hyperthermia. Therefore, it seems that the inhibition of HSP70 expression contributes to the main role of the mitochondrial pathway of apoptosis in cells exposed to hyperthermia in combination with ethanol, as compared with those only treated with hyperthermia. In fact, pharmacological inhibition of caspase-9 reduced modestly the percentage of apoptotic cells in response to hyperthermia while apoptosis was blocked in a greater extension when ethanol was present during the hyperthermia treatment.

The inhibitory effect of ethanol on HSP70 upregulation stimulated by hyperthermia was also observed in HL60 cells, an acute leukemia cell line in which ethanol also enhances hyperthermia-induced apoptosis. In addition, the effect of ethanol on hyperthermia-induced cell death was explored in other two human leukemia cell lines, MOLT3 and K562. The MOLT3 cells exhibit functional extrinsic and intrinsic apoptotic cell death pathways [75] and K562 cells contain the *Bcr-Abl* fusion gene and express high basal levels of HSP70 [35,76]. Ethanol failed to block HSP70 stimulation and did not increase the toxicity of hyperthermia in MOLT3 cells. These results rule out a non-specific effect of ethanol on the modulation of hyperthermia-induced apoptosis. We also found that K562 cells were highly resistant to hyperthermia and ethanol was unable to modulate this response. This is in accordance with the pro-survival role of HSP70 described in a number of tumors [77]. HSP70 gene transcription is regulated by the heat shock transcription factor 1 (HSF1), which becomes activated in response to hyperthermia among other stress stimuli [78]. Transcriptional activation of HSF1 is a complex process that sequentially involves translocation from the cytoplasm to the nucleus, trimerization, binding to the heat shock element (HSE) on DNA and phosphorylation [79]. It has been reported that ethanol is able to mediate activation of HSF1 and induction of HSP70 on human monocytes [80] and on cortical neurons [81,82]. On the contrary, catalase—which has been widely used as antioxidant—augmented the percentage of apoptotic cells in U937 cells subjected to heat, and decreased HSP70 expression through a mechanism involving HSF1 modulation [42]. In this context, it is interesting to note that ethanol under certain conditions may act as an indirect antioxidant [83] and that, in our conditions, there was not any evidence of oxidative stress by heat shock (Supplementary Materials, Figure S2). In consonance with these results, the antioxidants *N*-acetyl-L-cysteine (5 mM), glutathione (1 mM), allopurinol (10 µM), diphenyleneiodonium chloride (20 µM), and tiron (20 µM) were unable to block the effect of ethanol on apoptosis in cells exposed to hyperthermia (Supplementary Materials, Figure S3). Interestingly, the presence of ethanol during hyperthermia treatment was a requisite to achieve potentiation since there was not any effect when the alcohol was added at the beginning of the recovery period. These findings rule out non-specific effects and highly suggest that ethanol interferes in a step which is activated in the short time period (30 min) interval of hyperthermia treatment. These observations focus on HSF1 activation which is recognized to contain a sequence functioning as a quick temperature sensor [84]. It is accepted that the heat shock period corresponds to the moment in which HSF1 undergoes activation and binding to DNA, while the expression of HSP70 is achieved in the recovery period [42]. Further studies will be required to know whether ethanol modulates HSF1 activation and which step/s (if any) is modified.

## 4. Materials and Methods

### 4.1. Reagents

Protease inhibitors, 2′,7′-dichlorodihydrofluorescein diacetate (H2-DCF-DA), specific caspase inhibitors (z-DEVD-fmk, z-IETD-fmk, z-LEHD-fmk) and the colorimetric caspase substrates (Ac-DEVD-pNA, Ac-IETD-pNA and Ac-LEHD-pNA) were purchased from Sigma (St Louis, MO, USA). The pancaspase inhibitor z-VAD-fmk was obtained from Tocris (Bristol, UK). Polyvinylidene difluoride (PVDF) membranes were purchased from Millipore (Billerica, MA, USA). Acrylamide, bisacrylamide and the Bradford reagent were from Bio-Rad (Hercules, CA, USA). Antibodies against caspase-3 (Cat# ADI-AAP-113-F, RRID:AB_11180615), caspase-8 (Cat# ADI-AAM-118-E, RRID:AB_2038943) and caspase-9 (Cat# ADI-AAM-139-E, RRID:AB_2038946) were from Enzo Life Sciences. Antibodies against Bcl-2 (Cat# 2872, RRID:AB_10693462), Bcl-xL (Cat# 2764, RRID:AB_2228008), Mcl-1 (Cat# 4572, RRID:AB_2281980), Bax (Cat# 2772, RRID:AB_10695870), Bid (Cat# 2002, RRID:AB_10692485) and α-tubulin (Cat# 2125, RRID:AB_2619646) were from Cell Signaling Technology (Beverly, MA, USA). Antibodies against PARP (Cat# 551024, RRID:AB_394008), cytochrome c (Cat# 556433, RRID:AB_396417), Smac/DIABLO (Cat# 612246, RRID:AB_399569) and Beclin-1 (Cat# 612112, RRID:AB_399483) were from BD Biosciences (San Diego, CA, USA). Antibody against HSP70 (Cat# sc-24, RRID:AB_627760) and c-IAP1/2 (Cat# sc-12410, RRID:AB_2227909) were from Santa Cruz Biotechnology (Dallas, TX, USA). Secondary antibodies (Cat# NA9310, RRID:AB_772193; Cat# NA9340, RRID:AB_772191) were obtained from GE Healthcare (Little Chalfont, UK). Ethanol absolute was from Applichem GmbH (Darmstadt, Germany).

### 4.2. Cell Culture

Human leukemia cells U937 (DSMZ Cat# ACC-5, RRID:CVCL_0007), HL60 (DSMZ Cat# ACC-3, RRID:CVCL_0002), MOLT3 (DSMZ Cat# ACC-84, RRID:CVCL_0624), and K562 (DSMZ Cat# ACC-10, RRID:CVCL_0004) were grown in RPMI 1640 supplemented with 10% (*v*/*v*) heat-inactivated fetal bovine serum, 100 U/mL penicillin and 100 μg/mL streptomycin at 37 °C in a humidified atmosphere containing 5% CO_2_. Cell numbers were counted using a hematocytometer and the viability was >95% at the start of the experiments.

### 4.3. Exposure to Hyperthermia

At the beginning of each experiment the cells were resuspended with fresh growth medium and adjusted to 0.5 × 10^6^ cells/mL. The cells were treated with the specified percentage of ethanol, placed into sterile cell culture tubes and incubated for 30 min at 37 °C. Next, tubes were immersed for 30 min in a water bath previously adjusted at 37 °C (control) or 43 °C (hyperthermia treatment). Finally, the cells were transferred to cell culture plates, allowed to recover at 37 °C in a 5% CO_2_ atmosphere for the specified time periods, and harvested for biological analysis.

### 4.4. Cell Growth and Viability Assays

The cells were collected by centrifugation for 10 min at 500× *g* and washed with phosphate-buffered saline (PBS) at room temperature. After staining with a trypan-blue solution (0.4% *w*/*v* in PBS), the number of cells and the cell viability were determined with a TC10 automated cell counter (Bio-Rad, Hercules, CA, USA). Three samples per group were counted in each experiment and the experiments were performed at least three times.

### 4.5. Cell Cycle Analysis by Flow Cytometry

The cells were centrifuged at 500× *g* for 10 min at 4 °C, washed with 1 mL of ice-cold PBS, and fixed with 1 mL of ice-cold 75% ethanol at −20 °C overnight. The samples were then centrifuged and washed with ice-cold PBS. The cell pellets were resuspended in 200 μL of PBS containing 50 μg/mL propidium iodide and 100 μg/mL RNase A and incubated for 1 h at room temperature. The cell cycle phase distribution was analyzed by flow cytometry using a BD FACSVerse™ flow cytometer (Becton-Dickinson, Franklin Lakes, NJ, USA). A minimum of 10,000 cells per experimental condition were evaluated. Cell debris were excluded from analysis. The cells with decreased DNA staining (sub-G_1_ cells), resulting from either fragmentation or decreased chromatin, were considered apoptotic cells.

### 4.6. Annexin V–Propidium Iodide Double-Staining Assay

Apoptosis was also determined by analysing the translocation of phosphatidylserine to the cell surface using the annexin V-FITC apoptosis detection kit (BD PharMingen, San Diego, CA, USA), according to the manufacturer’s protocol. Briefly, cells were collected by centrifugation at 500× *g* for 10 min at 4 °C, washed twice with ice-cold PBS and then resuspended with annexin-binding buffer. A fraction of cell suspension (~50 μL) was incubated at room temperature for 15 min with 2.5 μL annexin V-FITC and 2.5 μL propidium iodide (50 μg/mL) and the samples were then diluted with 100 μL annexin-binding buffer and analyzed by flow cytometry.

### 4.7. Assay of Caspase Activity

The cells were collected by centrifugation at 500× *g* for 10 min at 4 °C, washed twice with ice-cold PBS, and lysed in caspase extraction buffer (50 mM HEPES [pH 7.4], 1 mM DTT, 0.1 mM EDTA, 0.1% Chaps) by pushing them through a 22-gauge needle. After centrifugation at 16,000× *g* for 10 min at 4 °C the supernatants were analyzed for protein concentration. Aliquots containing ~20 μg of protein were evaluated for caspase activity. Specific labeled substrates for caspase-3, caspase-8 and caspase-9 activities were N-acetyl-Asp-Glu-Val-Asp-p-nitroaniline (Ac-DEVD-pNA), N-acetyl-Ile-Glu-Thr-Asp- p-nitroaniline (Ac-IETD-pNA) and N-acetyl-Leu-Glu-His-Asp-p-nitroaniline (Ac-LEHD- pNA), respectively. Caspase-catalyzed release of the chromophore para-nitroaniline (pNA) from the substrate was measured at 405 nm in a microplate reader. Blanks containing the substrate alone were also included.

### 4.8. Immunoblotting

Treated cells were collected by centrifugation at 500× *g* for 10 min at 4 °C and washed twice with ice-cold PBS. To obtain cell lysates the cells were maintained on ice and resuspended with buffer A (20 mM Tris-HCl [pH 7.4], 2 mM EDTA, 137 mM NaCl, 10% glycerol, 1% Triton X-100) containing inhibitors of proteases (1 mM PMSF, 5 μg/mL of leupeptin, aprotinin and pepstatin A) and phosphatases (2 mM tetrasodium pyrophosphate, 2 mM sodium orthovanadate, 10 mM sodium fluoride, 20 mM sodium β-glycerophosphate). The lysates were sonicated on ice and centrifuged at 22,000× *g* for 15 min at 4 °C. The soluble fractions were used to determine the expression of caspase-3, caspase-8, caspase-9, PARP, Bid, BAX, Bcl-2, Bcl-xL, Mcl-1, cIAP1/2, HSP70 and α-tubulin. To prepare subcellular fractions, cells were kept on ice, resuspended in buffer B (20 mM HEPES [pH 7.4]), 1 mM EDTA, 1 mM EGTA, 1 mM DTT, 1.5 mM MgCl_2_, 10 mM KCl, 250 mM sucrose) supplemented with protease inhibitors as above and lysed with a 22-gauge needle. Lysates were centrifuged at 1000× *g* for 10 min at 4 °C to remove the nuclei. The resulting supernatants were centrifuged at 22,000× *g* for 15 min at 4 °C to obtain the cytosolic (soluble) fractions, used to determine cytochrome c, Smac/Diablo and α-tubulin. Whole cell lysates and cytosolic extracts were stored at –20 °C until used. Protein content was quantified by the Bradford method. Samples containing equal amounts of proteins were resolved by SDS-PAGE, transferred to PVDF membranes and then incubated with specific antibodies at 4 °C overnight. Blots were incubated with the appropriate horseradish peroxidase-conjugated secondary antibody and proteins detected by chemiluminescence (Millipore, Billerica, MA, USA). 

### 4.9. Analysis of Mitochondrial Membrane Potential

The cells (5 × 10^5^) were incubated with 10 μM of fluorescent probe JC-1 for the last 30 min of recovery, collected by centrifugation at 500× *g* for 10 min at 4 °C, washed with ice-cold PBS, and resuspended in 200 μL of PBS. This cationic and lipophilic probe accumulates and aggregates into active mitochondria of healthy cells, and aggregates emit fluorescence at 590 nm (red). Once mitochondrial membrane potential decreases, as occur upon cell injury, aggregates are transformed back into JC-1 monomers which emit fluorescence at 529 nm (green). Consequently, mitochondrial depolarization is indicated by a reduction in the red fluorescence. Cells were analyzed on a BD FACSVerse™ flow cytometer (Becton-Dickinson) using a 488 nm excitation with 527/32 nm and 586/42 nm bandpass emission filters. In each study, 10,000 cells were evaluated, and cell debris were excluded from analysis. Cells treated with 50 μM of the depolarizing agent carbonyl cyanide 3-chlorophenylhydrazone (CCCP) for 30 min were used as a positive control; in these conditions >95% of cells exhibited low mitochondrial membrane potential.

### 4.10. Intracellular Reactive Oxygen Species Determination

After treatment, the cells were further incubated with 10 μM of fluorescent probe 2′,7′-dichlorodihydrofluorescein diacetate (H_2_-DCF-DA, oxidation-sensitive by peroxides) or dihydroethidium (DHE, oxidation-sensitive by superoxide anion) for 30 min. The cells were collected by centrifugation at 500× *g* for 10 min at 4 °C, washed with ice-cold PBS and analyzed on a BD FACSVerse™ flow cytometer (Becton-Dickinson) using a 488 nm excitation with 527/32 nm (for detection of H_2_-DCF-DA oxidation) or 586/42 mm (for detection of DHE oxidation) bandpass emission filters. In each study 10,000 cells were evaluated, and cell debris were excluded from analysis.

### 4.11. Statistical Analysis

Data are presented as means ± SE. All determinations were performed in triplicate, and the data shown are representative results from at least three independent experiments. Statistical differences between means were tested using (i) Student’s t test (two samples) or (ii) one-way analysis of variance (ANOVA; three or more samples) with posteriori pairwise comparisons of means carried out using Tukey’s test. A significance level of *p* < 0.05 was used.

## 5. Conclusions

**Figure 7:**
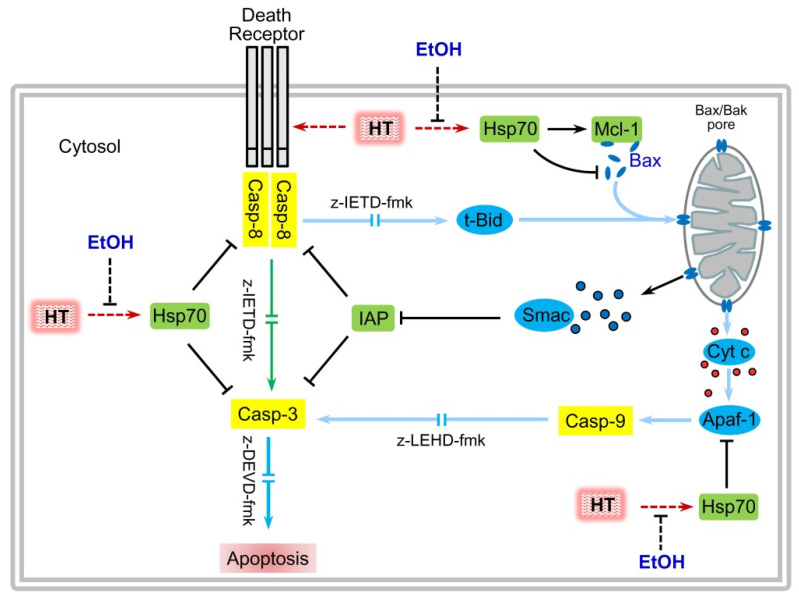
Ethanol enhances apoptotic cell death triggered by hyperthermia on human leukemia cells. Hyperthermia (HT) treatment alone stimulates apoptosis through a mechanism involving mainly caspase-8 and caspase-3 activities and increases the levels of the survival factor HSP70 which is able to: (i) stabilize the anti-apoptotic factor Mcl-1, (ii) to block Bax translocation and apoptosome formation (via binding to Apaf-1) and (iii) to inhibit caspase-8 and caspase-3. Ethanol abrogates hyperthermia-induced HSP70 expression, leading to an increase in caspase-8 activity, hydrolysis of Bid, translocation of Bax, release of mitochondrial factors (cytochrome c and Smac) and finally increase in caspase-9 and caspase-3 activities. Cyt c, cytochrome c; z-IETD-fmk, caspase-8 inhibitor, z-LEHD-fmk, caspase-9 inhibitor; z-DEVD-fmk, caspase-3 inhibitor; tBid, truncated Bid.

The data presented here allow us to conclude that ethanol enhances the effectiveness of hyperthermia to induce cell death on human leukemia cells through a mechanism dependent on caspase activation and associated with the decrease in the HSP70 protein (Figure 7). Because the effect of ethanol was achieved with non-cytotoxic concentrations, the results highly suggest its potential as a sensitizer of apoptosis in hyperthermia therapy for cancer.

## Data Availability

Data is contained within the article or Supplementary Materials.

## Supplementary Materials

The following are available online at https://www.mdpi.com/article/10.3390/ijms22094948/s1, Figure S1: Autophagy is not involved in the enhancement of hyperthermia-induced apoptotic cell death by ethanol, Figure S2: Ethanol does not stimulate reactive oxygen species generation in cells subjected to hyperthermia, Figure S3: Anti-oxidants do not block the enhancement of hyperthermia-induced apoptotic cell death by ethanol. Whole Western blots.
