# Supplementary material for: Ethanol Enhances Hyperthermia-Induced Cell Death in Human Leukemia Cells
Source: Int J Mol Sci. 2021 May 6;22(9):4948. doi: 10.3390/ijms22094948 (PMC8125413; doi:10.3390/ijms22094948)

Supplementary Materials

Figure S1: Autophagy is not involved in the enhancement of hyperthermia-induced apoptotic cell death by ethanol.

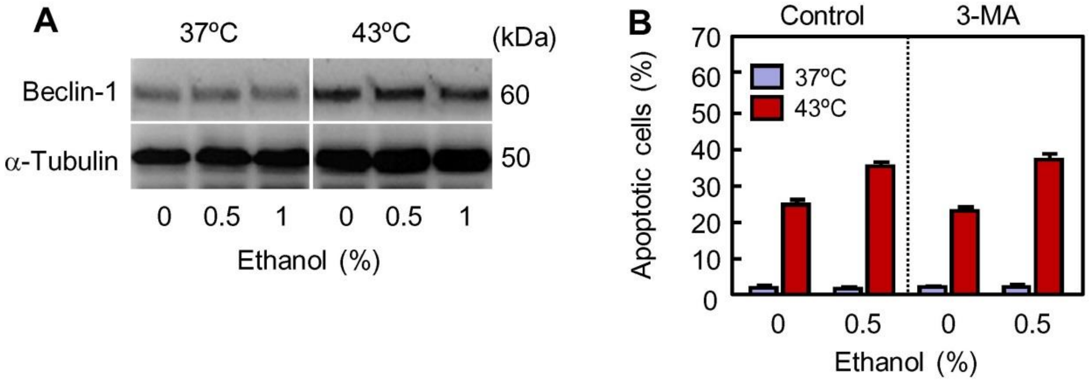

Figure S1. Autophagy is not involved in the enhancement of hyperthermia-induced apoptotic cell death by ethanol. U937 cells were pre-incubated with 3-methyladenine (3-MA, 5 mM) for 2 hr. The cells were then cultured in absence or presence of ethanol, exposed to hyperthermia (30 min at 43 ºC) and allowed to recover for 3 h. (A) Whole cell lysates were prepared and assayed by immunoblotting; as a loading control, α-tubulin was also determined. (B) The percentage of apoptotic cells was determined by flow cytometry using the propidium iodide staining procedure.

Supplementary Materials

Figure S2: Ethanol does not stimulate reactive oxygen species generation in cells subjected to hyperthermia.

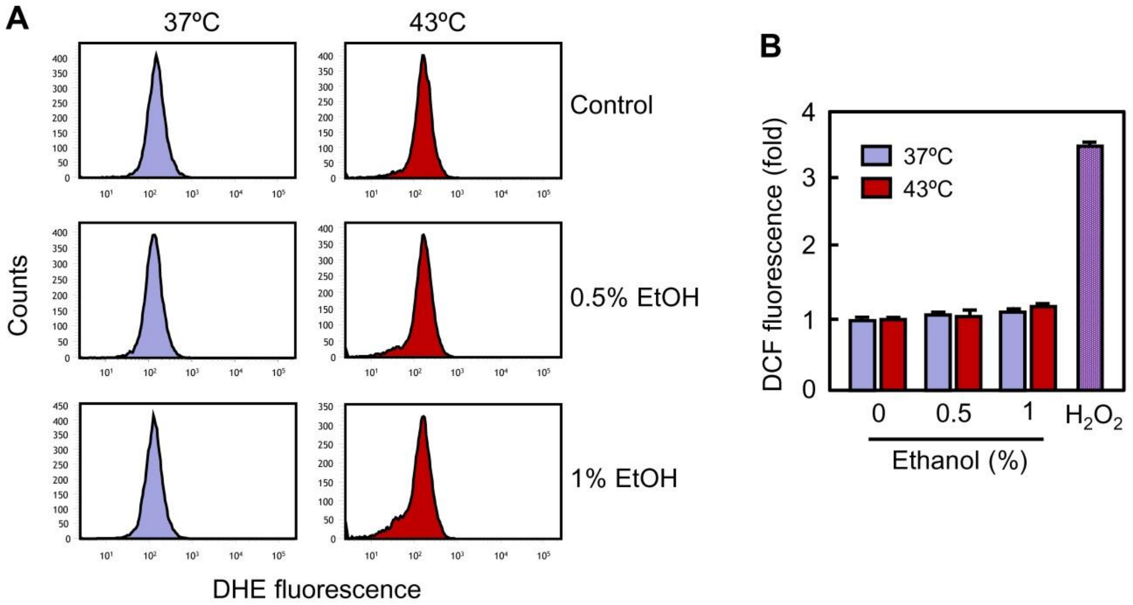

Figure S2. Ethanol does not stimulate reactive oxygen species generation in cells subjected to hyperthermia. U937 cells were pre-incubated in absence or presence of indicated concentrations of ethanol, exposed to hyperthermia (30 min at 43 ºC) and allowed to recover for 1 h. (A) Fluorescence of oxidized DHE was determined by flow cytometry as described in Materials and Methods section; histograms from a representative experiment are shown. (B) Fluorescence of oxidized DCF was determined by flow cytometry; means fluorescence from the resulting histograms were determined and represented as fold increase in comparison with cells cultured at 37 ºC in absence of ethanol. As a positive control, cells were treated with hydrogen peroxide (H_2O_2, 300 μM).

Supplementary Materials

Figure S3: Anti-oxidants do not block the enhancement of hyperthermia-induced apoptotic cell death by ethanol.

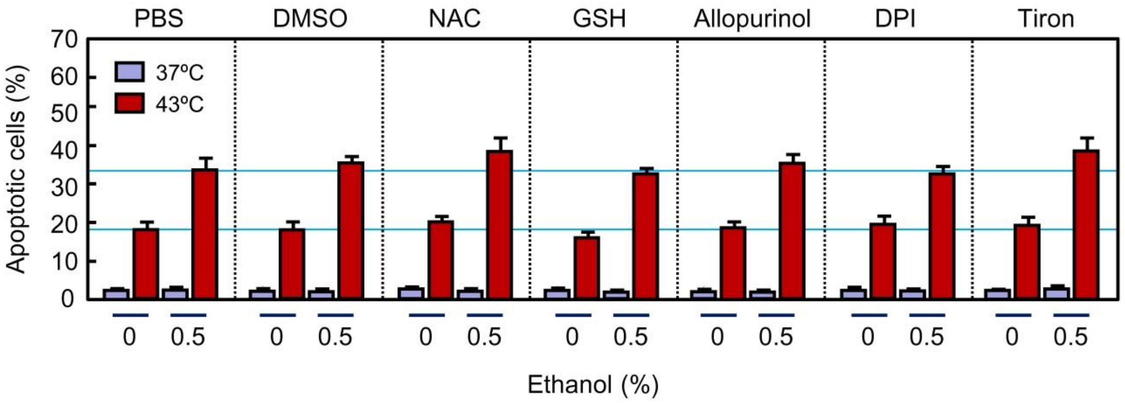

Figure S3. Anti-oxidants do not block the enhancement of hyperthermia-induced apoptotic cell death by ethanol. U937 cells were pre-incubated with *N*-acetyl-L-cysteine (NAC, 5 mM), L-glutathione (GSH, 1 mM), allopurinol (10 µM), diphenyleneiodonium chloride (DPI, 20 µM) or tiron (20 µM) for 2 hr. The cells were then cultured in absence or presence of ethanol, exposed to hyperthermia (30 min at 43 ºC) and allowed to recover for 3 h. The percentage of apoptotic cells was determined by flow cytometry using the propidium iodide staining procedure.

Supplementary Materials

Whole Western blots for Figure 4

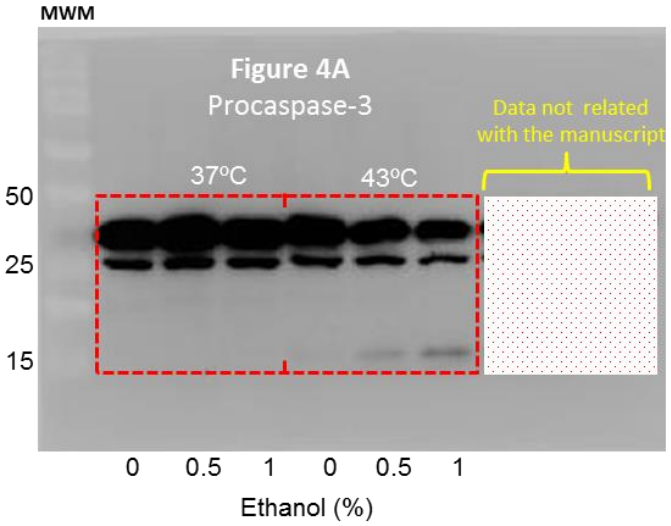

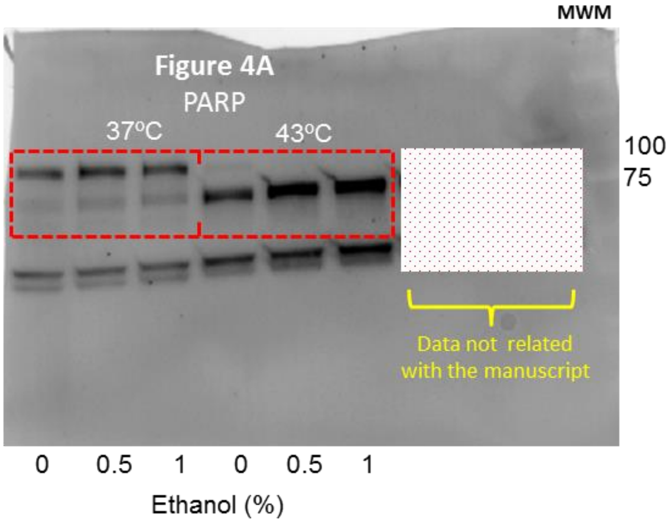

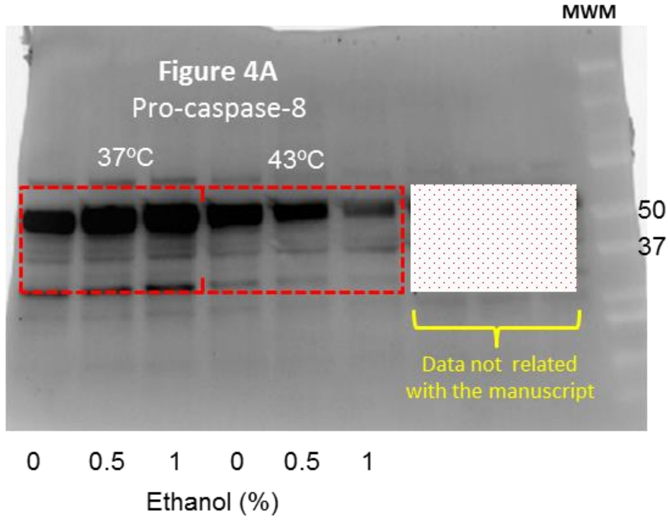

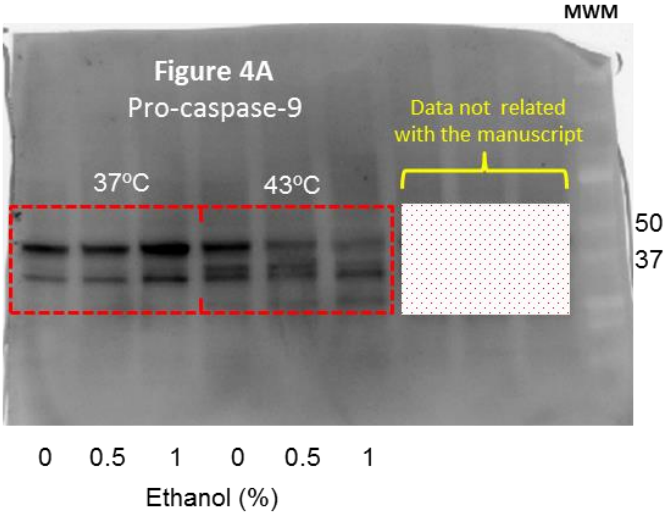

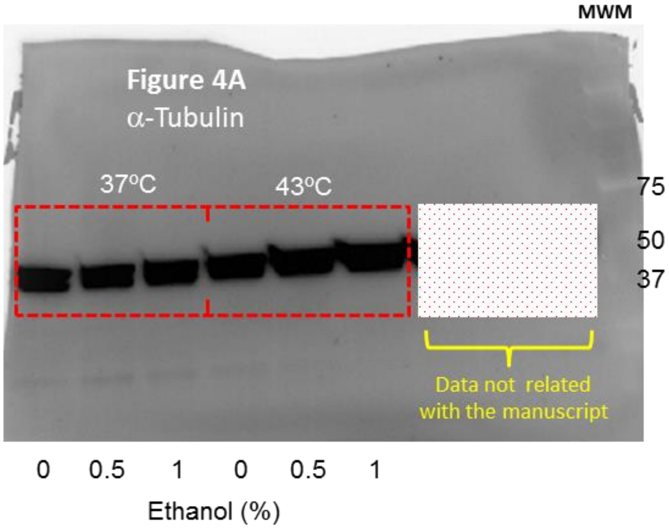

Supplementary Materials

Whole Western blots for Figure 5

Whole blot with molecular weight markers

Figure 5A

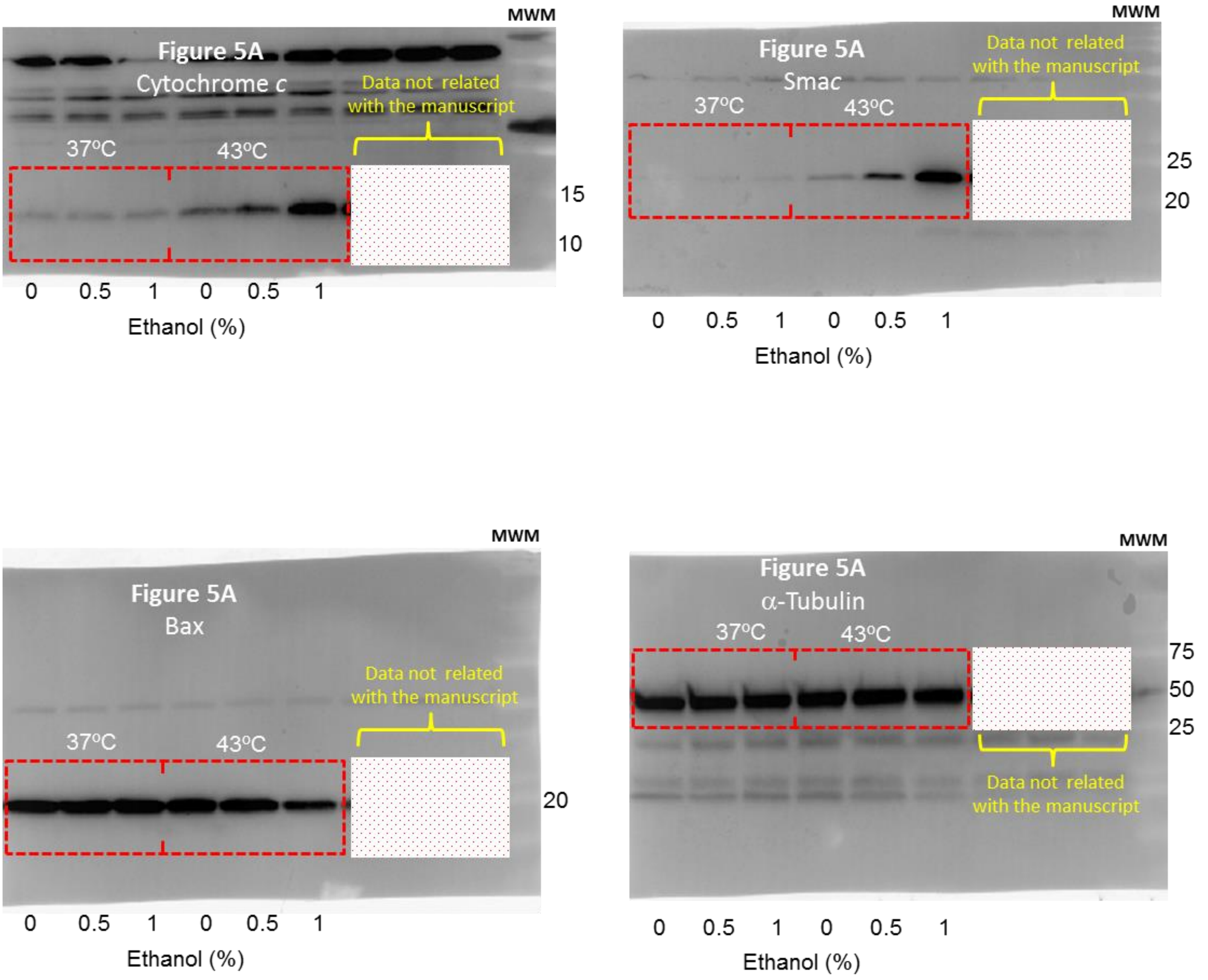

Supplementary Materials

Whole Western blots for Figure 5

Whole blot with molecular weight markers

Figure 5C

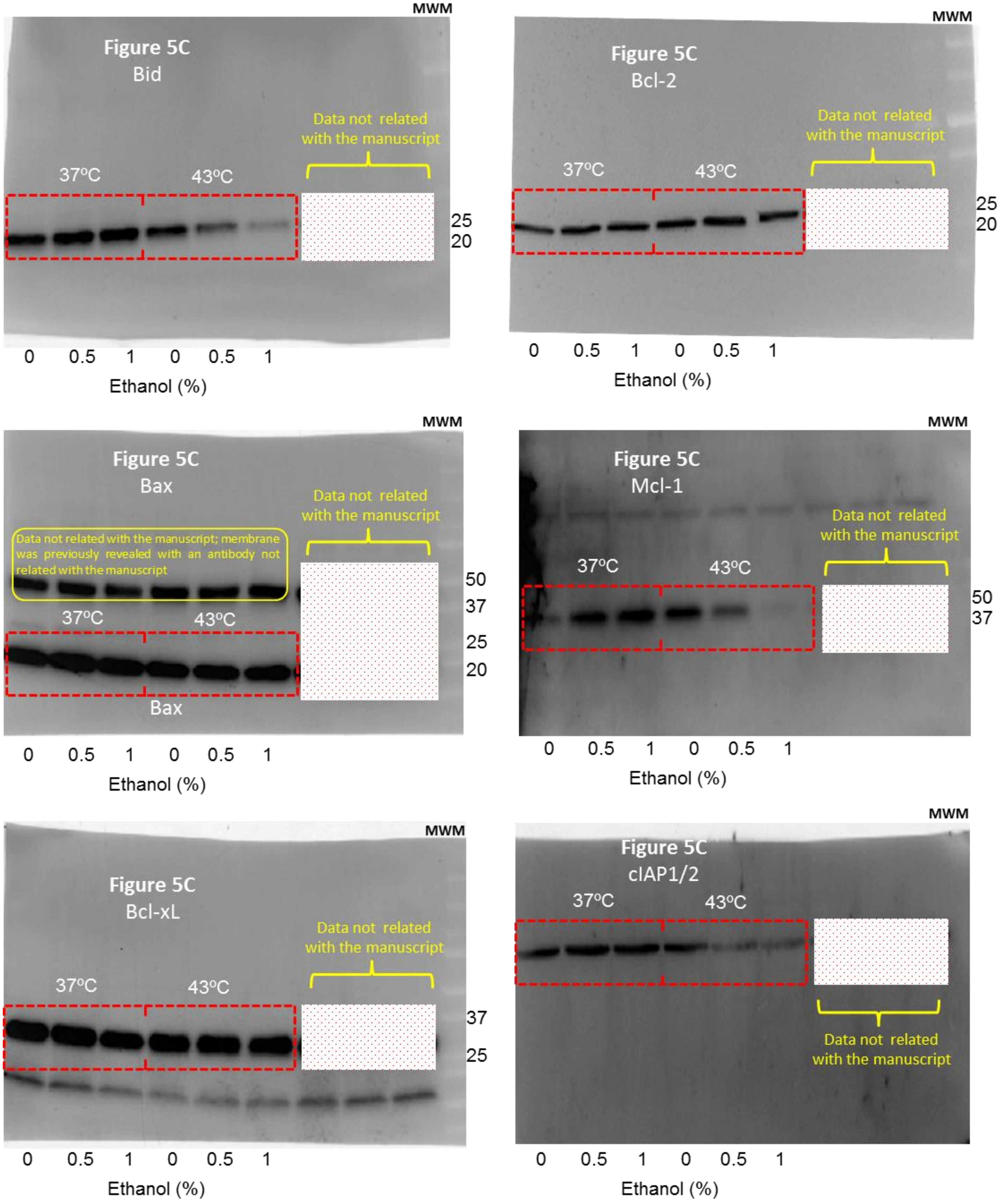

Supplementary Materials

Whole Western blots for Figure 5

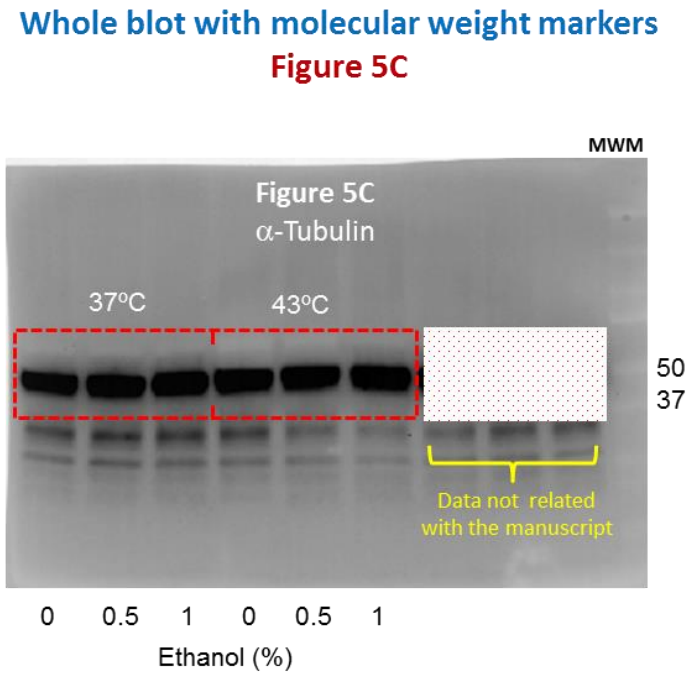

Supplementary Materials

Whole Western blots for Figure 6

Whole blot with molecular weight markers

Figure 6C

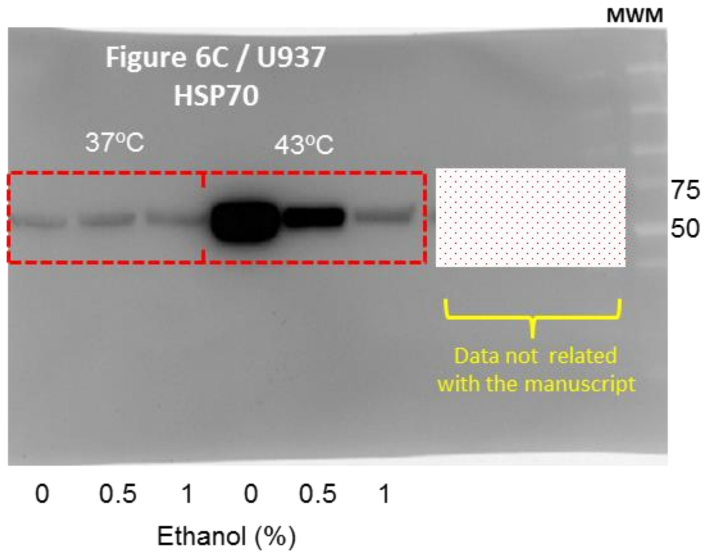

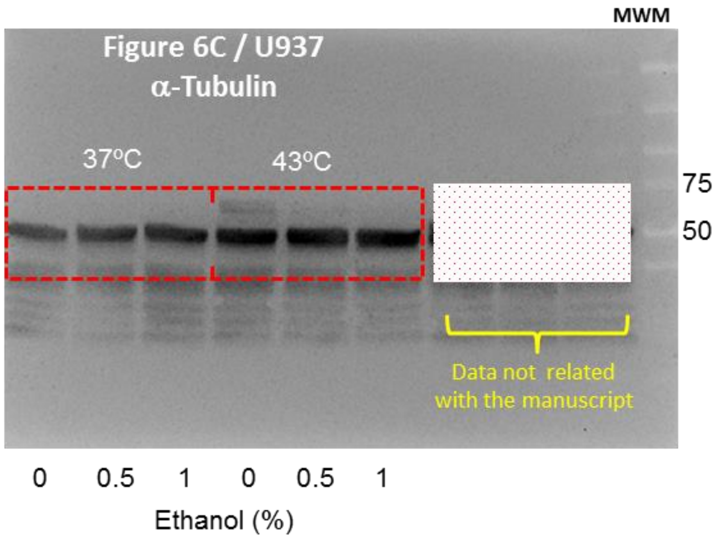

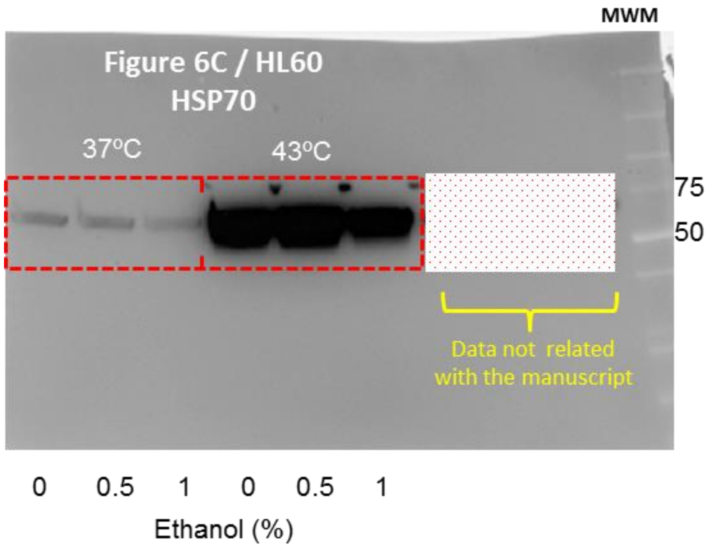

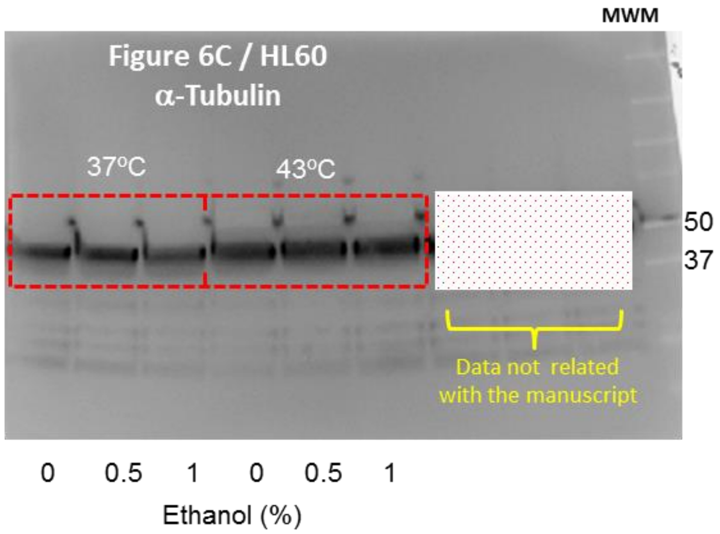

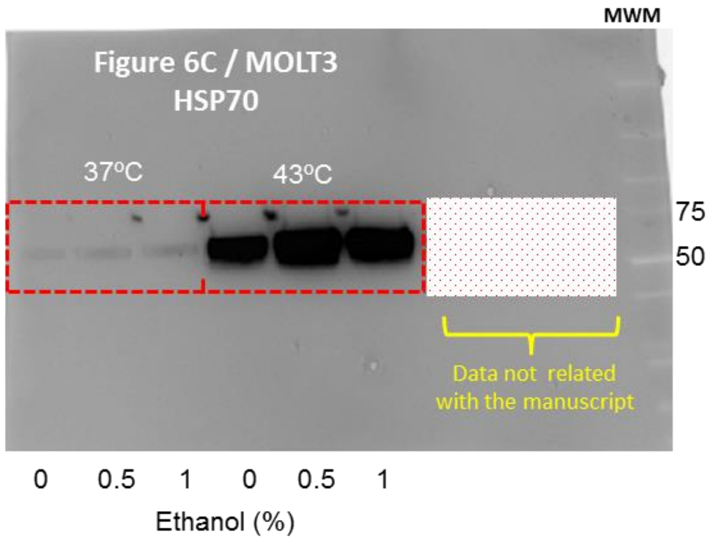

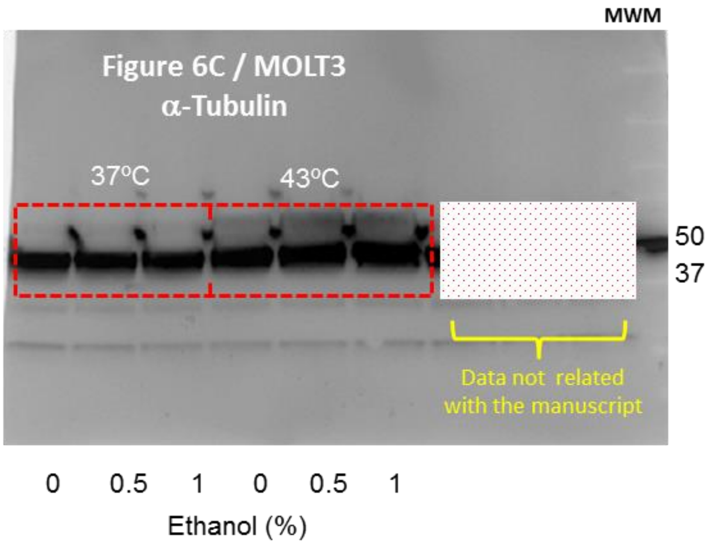

Supplementary Materials

Whole Western blots for Figure 6

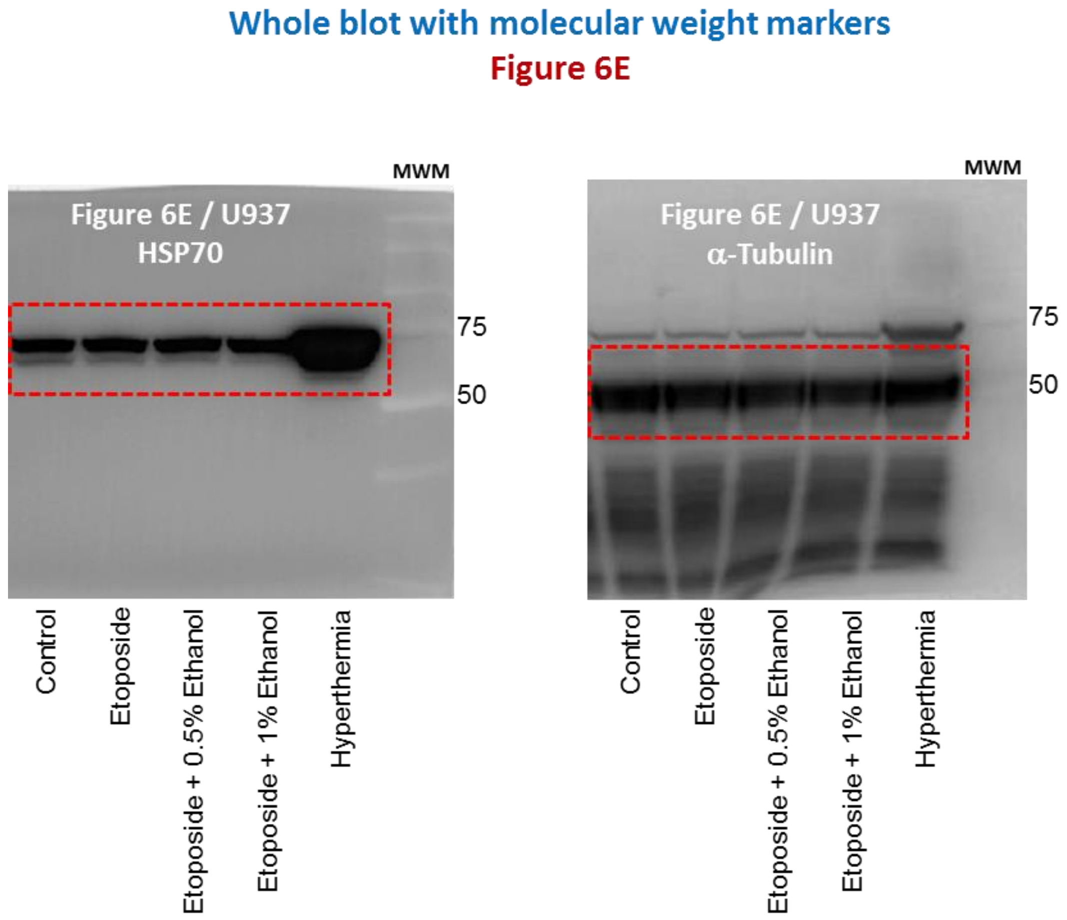

Supplement: Supplementary file 1 [file ijms-22-04948-s001.zip › ijms-1200370-supplementary.pdf]
